# A specialist surrounded by suburbia: the ecology of a newly described Four-toed Salamander population in New Brunswick

**DOI:** 10.7717/peerj.21370

**Published:** 2026-06-08

**Authors:** Ashton Michael James Leal, Georgia Arlene Christie, Julia Lindsay Riley, James Baxter-Gilbert

**Affiliations:** 1 Department of Biology, Mount Allison University, Sackville, New Brunswick, Canada; 2 Science Communication Unit, Imperial College London, London, United Kingdom

**Keywords:** Amphibian, Anthropogenic impact, Body condition, Conservation, Demographic, *Hemidactylium scutatum*, Imperilled species, Microhabitat, Urban ecology

## Abstract

**Background:**

Urbanisation can negatively impact biodiversity, often resulting in smaller and more isolated populations—which can be particularly detrimental for habitat specialists. Yet, under the right ecological conditions, urbanised greenspaces can also act as refugia for remnant populations to persist. The Four-toed Salamander (*Hemidactylium scutatum*) is an example of a macrohabitat specialist; it is an amphibian which relies specifically on habitat with bogs/fens and adjacent upland forests. In New Brunswick, Canada, this salamander is presumed to be critically imperilled as it had previously only been observed in a single location, a protected national park. A second population, however, was recently discovered within an urbanised greenspace in Riverview, New Brunswick. Notably, this area is enveloped by anthropogenic landscape features (*e.g.*, roads, parking lots, housing, businesses), which demonstrates how this species can also occur in more urbanised areas. Our study aims to understand the population ecology of Four-toed Salamanders at this newly described site and investigate how its habitat use allows it to persist in an area with strong anthropogenic disturbance.

**Methods:**

We estimated the current relative abundance and density, and quantified the demographics, of Four-toed Salamanders at the Riverview site, while also testing to see if these salamanders favoured specific microhabitats within both fen and upland forest habitats. We contrasted a wide range of suitable environmental variables (*e.g.*, substrate temperature, acidity, canopy cover, humidity, and plant percent coverage) between locations containing a salamander to nearby, unoccupied locations.

**Results:**

We found a total of 67 salamanders across all surveys, which translates to a conservative relative population density estimate of 2.12 individuals/ha. We observed few differences in microhabitats between where salamanders were seen and random locations, which suggests that suitable microhabitats were not limited for these amphibians at this site. These differences were that salamanders preferred higher slopes within moss hummocks in fens, cooler substrates in the forest, and the presence of woody debris in the forest.

**Discussion & Conclusion:**

Our study provides key insights into the habitat characteristics of Four-toed Salamanders at the northern extent of their range, a species with a cryptic lifestyle that is widely considered to be a macrohabitat specialist. By quantifying the ecological features of an urban habitat being used by Four-toed Salamanders in New Brunswick, we expand the types of ecosystems conservation biologists and wildlife managers should consider viable when conducting additional surveys and assessments for this species. This study increases our knowledge about the niche of an amphibian whose conservation and protection in New Brunswick, and more broadly in Canada, is hindered by a lack of foundational natural history data.

## Introduction

Biodiversity is declining at an alarming rate, requiring immediate conservation action (Barnosky et al., 2011); however, the lack of fundamental ecological information hampers such efforts (Borgelt et al., 2022). Major declines have been observed in amphibians (Luedtke et al., 2023), birds (Luna et al., 2018), insects (Brooks et al., 2012; Farriester et al., 2021), mammals (Zhao et al., 2024), and reptiles (Gibbons et al., 2000; Biber, Voskamp & Hof, 2023). As these declines accelerate, wildlife populations are becoming smaller and more isolated (Luna et al., 2018), making them susceptible to extinction vortices, wherein a lack of resources and increased genetic inbreeding depression continually worsens rapid population declines (Blomqvist et al., 2010; Benson et al., 2019; Williams et al., 2021). Anthropogenic activity is a leading factor impacting wildlife population declines and increases their isolation. Combinations of agriculture, climate change, overexploitation, pollution, and invasive species all contribute to escalating biodiversity loss (Isaksson, 2010; Medan et al., 2011; Tedeschi et al., 2022; Biber, Voskamp & Hof, 2023). One of the more prominent and increasingly pervasive factors, however, is the expansion of urban and suburban areas.

Urbanisation is a key factor degrading ecosystems and biodiversity as human populations continue to grow (Bai et al., 2019), often negatively impacting various wildlife populations (Ishitani, Kotze & Niemelä, 2003; Sanford, Manley & Murphy, 2009; Bozkurt & Basaraner, 2024). Generalist taxa who easily adapt to urban or suburban environments have a higher probability of surviving, and even thriving, in urbanised environments (Ishitani, Kotze & Niemelä, 2003; Sorace & Gustin, 2009; Ducatez et al., 2018). In contrast, species with specific habitat preferences have difficulty persisting in areas with increased urbanisation, as optimal breeding, feeding, and/or nesting habitat can be limited due to the homogenisation of landscapes during urban development (Grinnell, 1917; Clavel, Julliard & Devictor, 2011; Lemoine-Rodríguez, Inostroza & Zepp, 2020). Despite this, some specialist taxa can thrive in urban areas (Da Rocha-Filho et al., 2018; Stevens, Hale & Williams, 2023). For example, Swamp Rabbits (*Sylvilagus aquaticus*), which require mature forests with moderate flooding, can inhabit larger urban greenspaces when forest cover and water management enable its survival (Stevens, Hale & Williams, 2023). While urban development and sprawl results in habitat destruction, greenspaces with moderate human impact can create refuges for imperilled taxa to thrive, especially if these areas are protected from further development (Ives et al., 2016; Gallo et al., 2017; Zuñiga Palacios et al., 2021). Research into the ecological and evolutionary factors that permit or preclude wildlife from persisting in urban landscapes provides insight into the mechanisms that allow for ecological resiliency (Alberti, 2023). Yet, in the context of urbanisation, not all species are examined equally in the literature with less charismatic taxa, and/or taxa with cryptic lifestyles, often being overlooked.

Amphibians are one of the most imperilled groups of vertebrates, with 40.7% of 8,011 species being threatened with extinction (Luedtke et al., 2023; AmphibiaWeb, 2025). Within the class Amphibia, the order Caudata (*e.g.*, salamanders and newts) is the most imperilled order (Luedtke et al., 2023). Despite high imperilment, amphibians are also quite overlooked regarding conservation research and policy (Lawler et al., 2006; Donaldson et al., 2016). Many anthropogenic impacts coalesce within the process of urbanisation (*e.g.*, increased habitat degradation, fragmentation, and pollution), which are viewed as primary drivers of global amphibian biodiversity loss (Keely et al., 2015; Luedtke et al., 2023). However, exceptions exist where amphibians exploit urbanised landscapes and resources to persist or invade, like the Coquí Frog (*Eleutherodactylus coqui*), Eastern Red-backed Salamander (*Plethodon cinereus*), and Guttural Toad (*Sclerophrys gutturalis*) who capitalise on anthropophilic tendencies (Beard, Johnson & Shields, 2018; Wilk, Donlon & Peterman, 2020; Baxter-Gilbert et al., 2022). For example, Eastern Red-backed Salamanders can persist in urban greenspaces, with patch sizes not impacting genetic diversity in urban sites (1–250 ha) in Ohio, USA (Wilk, Donlon & Peterman, 2020), and can show higher abundances in urbanised populations compared to more rural ones in Atlantic Canada (Christie, 2024). In addition, the Northern Dusky Salamander (*Desmognathus fuscus*) and Southern Two-lined Salamander (*Eurycea cirrigera*) are stream salamanders that can persist in streams that are negatively impacted by urban development (Price et al., 2011; Rittenburg, Downing & Pierson, 2025). Significant knowledge gaps still exist regarding the factors that allow many amphibians, particularly native populations, to withstand urbanisation. In addition to these gaps, there is a research attention bias toward anurans, while salamanders remain overlooked due to their more secretive nature (Scheffers & Paszkowski, 2012).

The Four-toed Salamander (*Hemidactylium scutatum*) is a macrohabitat specialist with a cryptic lifestyle, requiring bog/fen habitats for nesting that are adjacent to upland forests where they feed and breed (Blanchard, 1923; Blanchard, 1934; McAlpine, 2010; Casebere & Lodato, 2011). When nesting, this species lays eggs in vegetation (typically within moss, especially *Thuidium delicatulum* and *Sphagnum* spp., hummocks) above open vernal pools of the bog/fen for their larvae to descend within and develop into adults (Chalmers & Loftin, 2006; Casebere & Lodato, 2011). This species has a patchy, often disjunct, distribution across its range, which has been suggested to be a result of their required habitat being previously more continuous and widespread during the post-glacial conditions of the Pleistocene (Herman & Bouzat, 2016). For example, since the 1980s only a single Four-toed Salamander population was documented in New Brunswick, Canada, within a national park; however, in 2023 a second population was discovered next to a high school in Riverview, New Brunswick, 59 km away from the first population (Christiansen et al., 2026). Based on the one known population found in the national park, the species is currently presumed to be critically imperilled in New Brunswick (NatureServe, 2026). Unlike previously described populations in North America (*e.g.*, Wood, 1955; Kilpatrick, 1997; Chalmers & Loftin, 2006; Moore, 2011; Vitale, 2013; King & Richter, 2022), this new population is enveloped by human development (*i.e.,* suburbia) and hosts various anthropogenic threats like recreational development, noise pollution, and refuse (Christiansen et al., 2026). Disturbance by predators may also be a threat to this newly found population because Raccoons (*Procyon lotor*), which are common in the area, have been documented to predate Four-toed Salamander nests in Tennessee, USA (Ferguson & Hamed, 2024). The existence of this new Four-toed Salamander occurrence, in a disturbed habitat at the northern extent of the species’ range, raised a couple key questions: (1) is this a viable population (*e.g.*, containing multiple adult females and males and is experiencing juvenile recruitment) and (2) how can a species thought to be a macrohabitat specialist survive in such an anthropogenically impacted habitat (*e.g.*, what habitat features allow this population to meet their ecological needs)? To answer these questions, we carried out a mark-recapture study while collecting morphometric and demographic data to estimate population ecology metrics. Concurrently, we examined Four-toed Salamander microhabitat preferences in fen and forest habitats by measuring an array of environmental factors (*e.g.*, substrate temperature, soil moisture, pH, plant percent coverage) and comparing these measurements to nearby locations lacking salamanders. We hypothesise that Four-toed Salamanders prefer microhabitat characteristics that mirror natural sites, predicting that individuals will select microhabitats that are moister, cooler, and have a denser overstory compared to random locations (Feder & Lynch, 1982; Harris, 2008; Casebere & Lodato, 2011; Wade et al., 2023). This study’s findings will increase our understanding of whether individuals are being challenged by anthropogenic pressures in this isolated urban forest (*i.e.,* holding on to highly specific microhabitat features within a broader matrix of unsuitable habitat), if Four-toed Salamanders here have a wider breadth of habitat preferences, or if the forest provides what they need, more so than previously expected.

## Materials & Methods

### Study site

**Figure 1:**
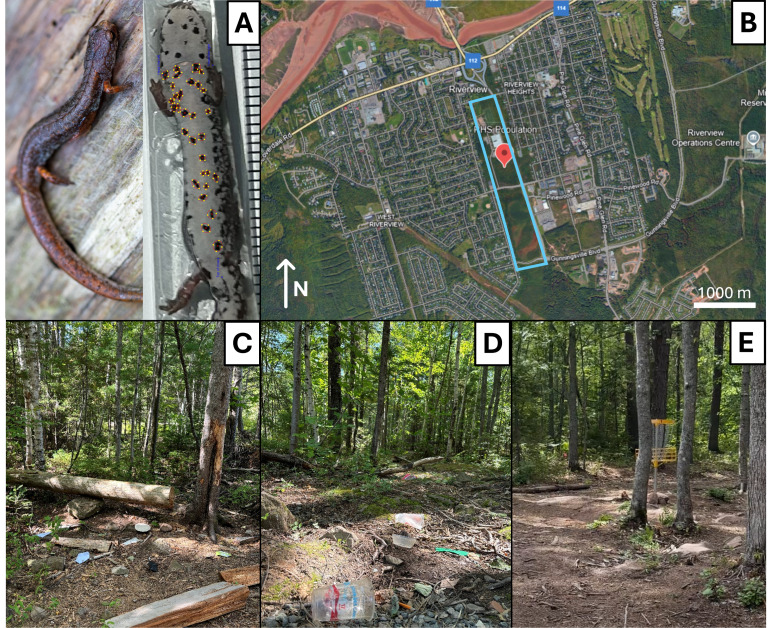
Riverview High School study site in Riverview, New Brunswick, Canada. (A) Dorsal view of a Four-toed Salamander captured during this study (picture taken by Joshua Christiansen) on the left that is paired with (on the right) a picture of the ventral side of a different salamander from the site annotated with reference points and ellipses from the pattern recognition software I^3^S Spot (*v.* 4.0.2). (B) Overview of the study site surrounded by suburban development created in Google Earth^®^ (accessed 1 April 2025; the map includes data from Google^®^ and imagery from 22 September 2017). The arrow points towards north. (C and D) Examples of anthropogenic disturbance in the Riverview High School study site highlighting litter. (E) Mulch laid down within the site to create human walking paths and a frisbee golf course.

The study site is 31.55 ha in size (measured using Google Earth^®^, *v.* 10.76.0.1) and is in Riverview, New Brunswick, Canada; it includes Riverview High School (RHS), as well as the adjacent land to the south (Figs. 1B–1E). The climate is temperate with daily average temperatures across this salamander’s active season (*i.e.,* April to October) ranging from 3.6 °C to 19.3 °C and precipitation of 678.7 mm. In contrast, the winter (*i.e.,* November to March) daily average temperatures range from −8.4 °C to 2.1 °C and the site receives 524.6 mm of precipitation (based on averages between 1991–2020; Environment and Climate Change Canada, 2025). The site sits 43 m above sea level (measured by a Garmin GPSMAP 66sr) and is comprised of a temperate mixed wood forest (*i.e.,* primarily birch, *Betula* spp., maple, *Acer* spp., pine, *Pinus* spp., and spruce, *Picea* spp.), footpaths for human access, a frisbee golf course (Fig. 1E), and several fens with sphagnum moss (*Sphagnum* spp.; Fig. S1). Approximately 19.5% of the site is fen habitat (6.14/31.55 ha; Fig. S1). The site is surrounded by anthropogenic development and disturbance, like paved roadways, parking lots, suburban housing, and shopping centres (Fig. 1B, Fig. S1).

### Data collection

To find Four-toed Salamanders, we conducted three different types of surveys (*i.e.,* road, fen, and upland) to increase our encounter rate. We undertook this multi-survey approach to counteract the typically low detectability of this species (Chalmers & Loftin, 2006). All individuals were handled under an animal ethics approval, which was provided by the Mount Allison Animal Care Committee (Animal Ethics Protocol # 103181) that follows the guidelines set out by the Canadian Council for Animal Care and under a scientific permit from the Government of New Brunswick (Permit #: SP24-006).

#### Road surveys

We walked roadways between 13 April to 10 July 2024 to detect if the fragmented nature of the urban landscape was requiring Four-toed Salamanders to cross roadways to migrate between habitat types. We surveyed, with 2–5 people, along a predetermined route around the Riverview site (Fig. S1A) after dusk during precipitation events (*e.g.*, ranging from mist to heavy rain), or when the roads were wet, and when air temperatures were above 5 °C (Mazerolle, Huot & Gravel, 2005; Clay & Gifford, 2017). When we found an individual, we collected morphometric and demographic data (see “Salamander Measurements” section for details).

#### Fen surveys

To search for Four-toed Salamanders within their nesting habitat, we surveyed four fen areas (Fig. S1B) in teams of 2–4 people between 15 May and 28 June 2024 during the daytime. These fens contained *Sphagnum* spp*.* moss that is optimal for nesting (Chalmers & Loftin, 2006; Casebere & Lodato, 2011; King & Richter, 2022). We surveyed the RHS fen three times completely and the other three fens twice completely (Fig. S1B). Survey teams moved along transects, spread approximately 4 m apart (*i.e.,* covering approximately 2 m on either side of each researcher), until the entire fen was surveyed. To locate Four-toed Salamanders and/or eggs, we would gently part large moss hummocks about 10 cm deep and hand capture individuals, and/or visually count clutches of eggs, as described by Chalmers & Loftin (2006), Casebere & Lodato (2011) and King & Richter (2022). We endeavoured to survey all moss hummocks in the fens to ensure we maximised the likelihood of encounters. We never surveyed a section of fen more than once within a 7-day period, as to not excessively disturb nesting salamanders or the habitat (Marsh & Goicochea, 2003).

#### Upland surveys

To encounter Four-toed Salamanders in their non-nesting habitat (*i.e.,* breeding, feeding, and overwintering), we examined sections of upland forest adjacent to the four fens, as well as a section of land south of Pinder Road due to its proximity to the most southern fen (Fig. S1C). Upland surveys took place opportunistically during the daytime between 19 June and 11 November 2024 in teams of 2–5 people. As with the fen surveys, each researcher was spread approximately 4 m apart (*i.e.,* covering approximately 2 m on either side of each researcher). We searched along transects of forest, overturning any cover object encountered that could create a suitable microhabitat, such as cool moist conditions (*e.g.*, logs, bark, rocks, branches, and other forms of debris) for salamanders, as described by Bishop (1941), Margenau et al. (2023) and Wade et al. (2023). Cover objects that would crumble or were too heavy to lift were left undisturbed to not disrupt or damage microhabitats. We surveyed transects until the entirety of the upland habitat was searched. The land directly adjacent to the RHS fen was searched twice completely, while the other areas were searched once completely. Additional upland surveys were completed the following autumn, from 28 August to 11 November 2025.

#### Salamander measurements

When we found a Four-toed Salamander, it was placed within a polyethylene bag (Freezer Medium, model 1789729; Ziplock^®^) that had the interior misted with deionised water. While it was in the bag, we measured snout-vent length (SVL) using a standard plastic ruler (± 0.1 cm). Juveniles were categorised using an SVL cut-off of ≤ 20 mm and were not sexed because they lack secondary sexual characteristics (Kilpatrick, 1997). Larger individuals (>20 mm) were considered sexually mature adults and were sexed. Sex was determined by secondary sexual characteristics examined with a magnifying lens (LED Loupe Triplet 10X-21 mm; Tekcoplus). Males have swollen nasolabial grooves, enlarged premaxillary teeth, and a squared snout (Bishop, 1941; Rucker et al., 2021). Females lack these traits (Rucker et al., 2021). After determining life stage and sex, we removed the individual from the bag and weighed it by placing it into a small plastic container, also misted with deionised water, on an electronic balance to the nearest 0.01 g (± 0.01 g, model UF-OG-200; Fuzion or model MG-EL-500-01-BK-PRO; Maxus). After the weight was recorded, we returned the individual to where it was captured and recorded that location using a handheld GPS unit (GPSMAP 66sr or GPSMAP 67; Garmin). We also recorded the locations of clutches of eggs without guarding females. Because the morphology of Four-toed Salamanders prevents candling (*i.e.,* their ventral skin is opaque) we could not determine if females were gravid (*i.e.,* we could not shine a light on their ventral surface to reveal the presence or absence of eggs).

#### Mark-recapture

While each individual was within the polyethylene bag, we photographed their ventral surface against a white background (*e.g.*, a small whiteboard) with a smartphone camera to identify individuals. Four-toed Salamanders have unique spotting patterns on their venters that identifies an individual throughout life (Harris, 2008). We inputted these photographs into a pattern matching software (I^3^S Spot, *v.* 4.0.2) to identify recaptures (Den Hartog & Reijns, 2014). Thirty spots were annotated on each salamander by drawing closely fitted ellipses around the spots within and around a triangular reference area in the program (Fig. 1A). I^3^S Spot only allows users to add up to 30 ellipses, as adding more than 30 may negatively impact the program’s ability to identify recaptures (Den Hartog & Reijns, 2014). If there were <30 spots on a salamander’s ventral surface, we would annotate all defined spots (*n* = 1/70). We annotated each salamander’s stomach photograph (*n* = 70), added them to a database, and compared each photograph to the first 10 matches when searched. Given that paired photographs are likely to be identified within the first 10 matches provided by the I^3^S Spot software, we visually examined the first 10 potential matches to determine if a recapture photo correctly aligned with a known individual in the database (*i.e.,* confirming a recapture; Den Hartog & Reijns, 2014; Gardiner et al., 2014). The efficacy of the I^3^S Spot software with our photographs was tested by including replicated photographs of five Four-toed Salamander individuals, which were all taken at slightly different angles, that we knew were already in the database. We then assessed whether these replicated photographs would be identified within the first 10 matches. For all five of these individuals, their match was indeed identified within the first 10 matches—typically, as the first or second suggested individual.

#### Environmental measurements

We only took environmental measurements during fen and upland surveys, as we interpreted roads were not an environment that salamanders were favouring or requiring, but rather a habitat feature they simply needed to move across. In both fen and upland surveys, we estimated percent coverage by plant type (*i.e.,* woody plants, herbaceous plants, trees, leaf litter, and woody debris) within a 50 × 50 cm quadrat (2,500 cm^2^ area) centred where we found a salamander or clutch(es) of eggs. If sections of the quadrat area were not covered by plant material, we estimated the percentage of open soil substrate for those sections. We would take substrate temperature (°C) immediately upon observing a clutch of eggs and/or capturing a salamander with an infrared digital thermometer (± 0.1 °C, model 057-4554-4, Mastercraft) and canopy cover in % coverage with a convex spherical densiometer (± 0.01%, Model-C, Forest Densiometers).

At each point where a Four-toed Salamander was found in fen habitat, we took a series of additional environmental measurements. Depth (cm) to the bottom of the substrate (*i.e.,* the depth of loose surface debris and/or vegetation) was taken with a standard ruler (± 0.1 cm). The acidity of water was measured with a water pH strip (± 0.3, model BDH83932.601, VWR; ± 1, model BDH35309.606, VWR). Water temperature (°C) was recorded with a digital, metal probe thermometer (± 0.1 °C, model 057-4554-4, MasterChef). The slope (°) of nesting, or potential nesting, habitat was taken by measuring the inclination angle of the moss hummocks using a spirit level (± 1°, model 43-619, FatMax Xtreme Torpedo Level; Stanley). At each point where a Four-toed Salamander was found within upland habitat, we took another set of additional environmental measurements. Soil acidity was measured with an electronic pH meter (± 0.1, model 94302; Kel Instruments) and soil moisture was quantified with an electronic tensiometer (± 0.01%, SM 150 Soil Moisture Kit; Hoskin).

Once environmental data were collected at a location where a salamander was found, either in the fen or upland habitat, we took the corresponding environmental measurements at a paired, but random, location. We used a random number generator to set a direction (*i.e.,* a number range between 0 and 359, representing all directions within a 360° rotation with reference points at 0° = North, 90° = East, 180° = South, and 270° = West), as well as distance (*i.e.,* a number range between 3 and 30, representing meters to travel). At each random location, we set a 50 cm × 50 cm quadrat (2,500 cm^2^ area) in place and thoroughly searched the area within it for Four-toed Salamanders to ensure no individuals were present. If no individuals were found, we would repeat the environmental measurements, as appropriate for the survey type, here. If the random distance and direction brought us to the edge of generally suitable habitat (*e.g.*, reaching the edge of a forest or fen we were surveying) we travelled back in the opposite direction for the remaining number of meters we were required to travel, continuing past the point as necessary.

### Data analysis

All data analysis and visualisation were conducted in R (*v.* 4.4.2, R Core Team, 2024). Plots were generated using the packages ‘*ggplot2*’ (*v.* 3.5.1, Wickham, 2016) and ‘*gghalves*’ (*v.* 0.1.4, Tiedemann, 2022). The ‘*autoplot*’ function in ‘*ggfortify*’ (*v.* 0.4.17, Tang, Horikoshi & Li, 2016) was used for the principal component analysis visualisations. An α of 0.05 was selected for this study to denote statistical significance. All data is summarised as a mean ± standard error. All *t*-tests were completed using the ‘*t.test*’ function in the ‘*stats*’ package (*v.* 4.4.2; R Core Team, 2024) in accordance with Logan (2012). If data were not normally distributed, tested with a Shapiro–Wilk normality test, and/or the data was not homogenous, tested with a Levene’s test, we attempted transformations to fix the non-normal and/or non-homogenous data. If transforming the data did not achieve normality and or homogeneity, we performed a non-parametric equivalent of the desired model (*e.g.*, a Wilcoxon signed rank test using the ‘*wilcox.test*’ function in the ‘*stats*’ package (v. 4.4.2; R Core Team, 2024)) instead of a paired *t*-test (Logan, 2012).

#### Population status and demography

In this study, we estimated relative abundance (*i.e.,* by sampling to estimate true abundance of salamanders within this population of Four-toed Salamanders, and thus the estimate is dependent on our survey methods; Callaghan et al., 2024) which we define as the number of individuals present at the substrate surface and observed during our surveys, similar to Price et al. (2006), Otto, Roloff & Thames (2014) and Romano et al. (2017). We also estimated the relative density of salamanders at the site, which was calculated by dividing our measure of relative abundance by the area of the study site in hectares. The distribution of salamanders was visualised by plotting each individual location on Google Earth^®^ (*v.* 10.76.0.1; Fig. S3). We collated the demographic data, compared the number of males to females (*i.e.,* M:F) and reported how many juveniles were found.

To test for morphological differences between adults, we applied an unpaired *t*-test between the SVL of females and males to determine the presence and degree of sexual size dimorphism in the population. Additionally, we examined the body condition of females and males with the scaled mass index (SMI), which is derived from a standardised major axis (SMA)-adjusted ordinary least squares (OLS) regression of log-transformed body mass on log-transformed SVL (Peig & Green, 2009). This method accounts for allometric scaling which provides a scaling exponent that adjusts mass to a standardised SVL to make body condition estimates more size-independent (Peig & Green, 2009). Higher SMI values are assumed to indicate better, or ‘healthier’, body conditions with more energy/fat stores in an animal compared to an animal with a lower SMI value. SMI can also be calculated with a robust regression model to better account for data outliers (Maronna et al., 2019). Thus, we compared using OLS and robust regression estimators to assess which method best described the mass–SVL relationship for the Four-toed Salamanders in our population. After visually assessing the OLS and robust regression trendlines on the data (see Fig. S2) we determined that the robust regression better fit the data. We then compared SMI between females and males *via* the robust regression residuals using a Wilcoxon rank sum test with a continuity correction, due to failed normality, to determine whether females or males were in a better, or ‘healthier’, body condition compared to the other sex.

#### Microhabitat preferences

To examine if Four-toed Salamanders prefer specific microhabitats, we completed a paired *t*-test or the nonparametric equivalent (*i.e.,* a Wilcoxon signed rank test with a continuity correction) on each environmental variable except for plant percent coverage by type. To examine the upland environmental and plant type data in an alternative way, we used a principal component analyses (PCA) to summarise the variables using the ‘*princomp*’ function in the ‘*stats*’ package (*v.* 4.4.2; R Core Team, 2024) and then compared these variables between locations with salamanders and random locations. We retrieved eigen values using the ‘*get_eigenvalue*’ function in the ‘*factoextra*’ R package (*v.* 1.0.7; Kassambara & Mundt, 2020). We selected eigen values above 1 to be examined further *via* data visualisation using the ‘*autoplot*’ function in the ‘*ggfortify*’ R package (*v.* 0.4.17; Tang, Horikoshi & Li, 2016). Lines of data with missing values had to be removed from the PCA so we could run that model. This did not impact the plant type PCA but did impact the environmental variable PCA with 53 out of 62 total data points (*i.e.,* salamander and random data points combined) being included. Principal component loadings were produced using the ‘*loadings*’ function in the ‘*stats*’ package (*v.* 4.4.2; R Core Team, 2024). We then compared the PC1 and PC2 values between the salamander and random treatments with two-sample *t*-tests or their nonparametric equivalent (*i.e.,* a Wilcoxon rank sum test with a continuity correction) for both PCAs.

## Results

### Population status and demography

We encountered a total of 42 Four-toed Salamanders during our surveys in 2024, and 29 in 2025 (see Fig. S3). Four salamanders were located during road surveys, seven during fen surveys, and 60 during upland surveys. Of these 71 salamanders encountered, we have individual-based data on 67 unique salamanders (*i.e.,* not included one recapture, two deceased, and one escapee pre-identification; Table 1). This relative abundance of 67 salamanders was used to calculate a relative density estimate within the entire study site, which is 2.12 individuals/ha. One of the deceased individuals was a specimen euthanised and taken by the New Brunswick Museum for their genetic database in 2024 and the other was found dead under a cover object in a spider web in 2025. The recaptured individual, an adult male, was identified by I^3^S Spot and a manual visual assessment corroborated this finding.

**Table 1:** The number of Four-toed Salamanders (*Hemidactylium scutatum*) captured in Riverview, New Brunswick, Canada, from 2024 and 2025 by survey type, separated by life stage and sex. The number of clutches of eggs observed are also noted. The table excludes one re-captured, two deceased individuals, as well as one individual that escaped pre-identification. The sex of juveniles (*i.e.*, individuals ≤ 20 mm) could not be identified as they do not yet express secondary sex characteristics. Only upland surveys occurred in 2025.

**Year**	**Survey type**	**Males**	**Females**	**Juveniles**	**Unknown**	**Total**	**Egg clutches**
2024	Road	1	3	0	0	4	NA
	Fen	0	4	2	0	6	4
	Upland	9	15	6	0	30	NA
2025	Upland	13	13	0	1	27	NA
**Column Total**	NA	23	35	8	1	67	4

In 2024, we found 10 males, 23 females, and eight juveniles, which led to an adult M:F sex ratio of 1:2.3. This summary includes a bias towards females, however, due to the inclusion of salamanders found during fen surveys (predominately occupied by females) and road surveys (primarily females likely migrating between breeding and nesting habitat). So, separately considering only the salamanders found during upland surveys, the adult M:F sex ratio was 1:1.8 (from nine males, 16 females, with an additional six juveniles observed). In 2025, we found a total of 15 males, 13 females, zero juveniles, and one salamander where the sex was unknown, which led to a M:F sex ratio of 1:0.9 and no female skew. With both years combined, which is including living males (accounting for the recaptured individual), females, and juveniles we found in 2024 and 2025 (23 males, 35 females, and eight juveniles; Table 1) we get a combined adult M:F sex ratio of 1:1.5.

During our fen surveys, we located five clutches of eggs. Two of these clutches were near each other (*i.e.,* there were two defined groupings of eggs in the same moss hummock with a female). One clutch of eggs was found in the southernmost fen, while the remaining four were found in the RHS fen (see Fig. S3). Out of the four individuals found during road surveys, three were observed on a gravel shoulder and one was found on the entrance road to RHS. These individuals were all observed alive and so no evidence of road mortality from road crossings was observed.

**Figure 2:**
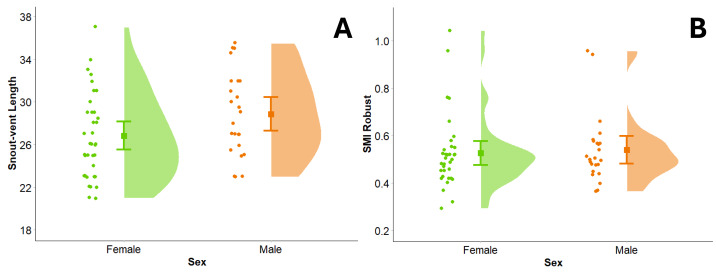
Sexual dimorphism between Four-toed Salamander (*Hemidactylium scutatum*) females (green) and males (orange). (A) A plot of the snout-vent length (mm) for each individual (*t*_58_ = −2.041, *p* = 0.046; two-sample *t*-test). (B) Plot of the residuals from a scaled mass index robust regression (SMI Robust) of mass (g) *vs.* snout-vent length (mm) for each individual (*W* = 388.5, *p* = 0.632; Wilcoxon rank sum test with a continuity correction). The square points represent means, the error bars are 95% confidence intervals, the points on the left of the error bars are raw data points, and half violin plots are on the right of the error bars.

Sexual size dimorphism was observed with males being significantly longer than females, based on SVL (females: 26.7 ± 0.7 mm; males: 28.9 ± 0.8 mm; *t*_58_ = −2.041, *p* = 0.046; two-sample *t*-test; Fig. 2A). Despite the difference in body length, there were no significant differences in SMI with a robust regression model between the sexes (females: 0.53 ± 0.03 g; males: 0.54 ± 0.03 g; *W* = 388.5, *p* = 0.632; Wilcoxon rank sum test with a continuity correction; Fig. 2B).

### Microhabitat preferences

#### Fen Habitat

We observed that in fen habitats, Four-toed Salamanders were located, and eggs were more consistently laid, in moss hummocks that had steeper slopes compared to random paired locations (*t*_7_ = 2.475, *p* = 0.043; paired *t*-test), favouring a mean slope of 35.5 ± 7.1° where salamanders and/or eggs were compared to 14.8 ± 3.6° at random locations (Table 2; Fig. S4A). Although the mean water temperature (°C) between salamander and random locations was not significantly different (*t*_7_ = −2.030, *p* = 0.082; paired *t*-test), salamanders were found at water temperatures of 16.2 ± 0.5 °C whereas random location water temperatures were an average of 18.5 ± 1.0 °C (Table 2; Fig. S4B). Salamanders appeared to favour a more restricted temperature range, which is supported by the fact that the coefficient of variation (CV) of water temperatures documented at locations with salamanders was 8.1. This is approximately two times less than the CV of water temperatures recorded at random locations, which was 16. The rest of the environmental variables did not significantly differ, either statistically or biologically, between random locations and those that contained salamanders, including fen substrate temperature (*V* = 8.5, *p* = 0.109; Wilcoxon signed rank test with a continuity correction), substrate depth (*t*_8_ = 1.338, *p* = 0.218; paired *t*-test), water pH (*V* = 10.5, *p* = 1.000; Wilcoxon signed rank test with a continuity correction), and canopy cover (*V* = 13.0, *p* = 0.301; Wilcoxon signed rank exact test) (Table 2; Figs. S4C–S4F).

**Table 2:** Environmental measurements based on survey type (ST) and location type (LT) with literature comparisons ± standard error (unless otherwise specified; SD, Standard Deviation). Ranges are provided beneath standard error values within brackets. Sub T., Substrate Temperature (°C); Canopy, Canopy cover (%); Slope, Slope of the substrate (°); Water T., Water Temperature (°C); Sub D., Substrate Depth (cm); Soil M., Soil Moisture (%). We also include *p*-values from the comparisons between salamander and random point means *via* paired *t*-tests or the non-parametric equivalent. The “Literature” row shows the range of means (if more than one paper) or the mean ± standard error or SD presented in the literature for a given environmental variable where Four-toed Salamanders have been found. The “Source” row provides the source(s) for the listed literature values.

**ST**	**LT**	**Sub T.**	**Canopy**	**Slope**	**Water T.**	**Sub D.**	**Water pH**
Fen	Salamander	20.2 ± 1.3 (11.3–23.0)	30.7 ± 10.6 (0.52–72.44)	35.5 ± 7.1 (4.0–65.0)	16.2 ± 0.5 (13.9–18.0)	13.2 ± 3.0 (0–26.5)	5.8 ± 0.1 (5.4–6.6)
	Random	22.9 ± 1.0 (17.1–26.4)	46.6 ± 10.9 (0.78–89.08)	14.8 ± 3.6 (0.0–30.0)	18.5 ± 1.0 (15.0-22.7)	9.6 ± 2.4 (1.0–24.7)	5.8 ± 0.1 (5.4–6.3)
	***p*** **-value**	0.109	0.301	0.043	0.082	0.218	1.000
	**Literature**	18.1–33.0	13 ± 33 (SD)	43.33–75.4	13.5–28.0	11.22 ± 5.33 (SD)	5.0–6.3
	**Source(s)**	Wood (1955) and Kilpatrick (1997)	Chalmers & Loftin (2006)	Chalmers & Loftin (2006) and Wahl, Harris & Nelms (2008)	Wood (1955) and Kilpatrick (1997)	Chalmers & Loftin (2006)	Wood, (1955) and King & Richter (2022)
**ST**	**LT**	**Sub T.**	**Canopy**	**Soil M.**	**Soil pH**		
Upland	Salamander	18.6 ± 1.0 (4.4–29.5)	88.0 ± 2.1 (41.70–99.48)	16.9 ± 1.9 (5.2–43.4)	6.5 ± 0.1 (5.6–7.1)		
	Random	20.3 ± 1.1 (4.5–28.6)	85.3 ± 2.1 (44.80–97.14)	19.3 ± 2.1 (4.8–46.9)	6.3 ± 0.1 (5.5–7.0)		
	***p*** **-value**	<0.001	0.170	0.397	0.075		
	**Literature**	NA	NA	NA	3.3–7.45		
	**Source(s)**	NA	NA	NA	(Kilpatrick, 1997; acid–base tolerance range)		

#### Upland Habitat

**Figure 3:**
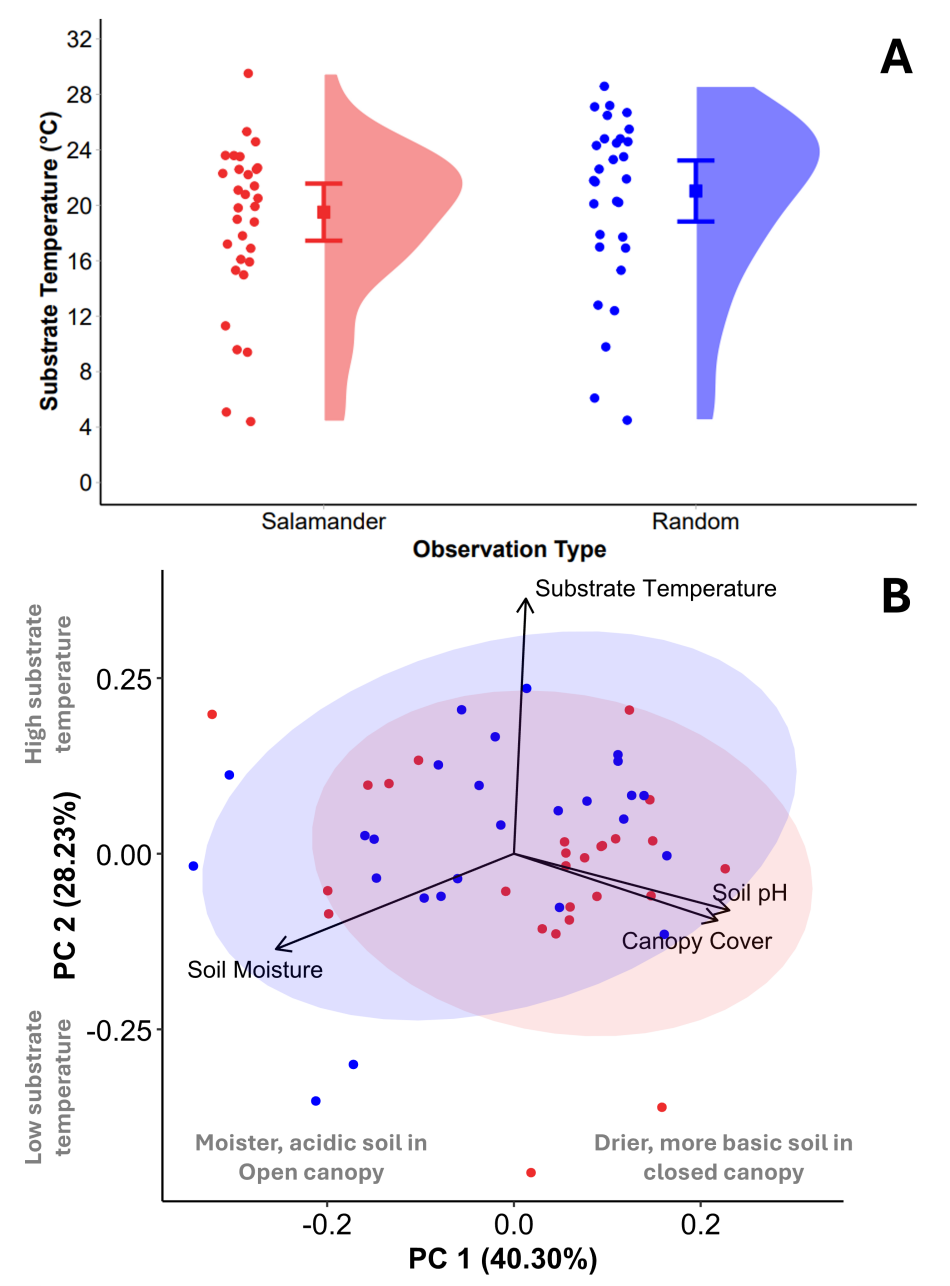
Plots of upland environmental variables recorded where Four-toed Salamanders (*Hemidactylium scutatum*) were found (red) and at paired random locations (blue). (A) Substrate temperature (°C) recorded at each location (*V* = 45, *p* = 0.001; Wilcoxon signed rank test with a continuity correction). The square points are means, the error bars are 95% confidence intervals, the points on the left of the error bars are raw data, and half violin plots are on the right of the error bars. (B) Principal component analysis visualization. Principal component one (PC1) is plotted along the *x*-axis explaining 40.30% of the total variance. PC1 reflects variation in soil moisture, soil pH, and canopy cover with loadings of −0.626, 0.567, 0.535, respectively. Principal component two (PC2) is plotted along the *y*-axis explaining 28.23% of the variance. PC2 reflects variation in substrate temperature with a loading of 0.892. The 95% confidence ellipses encapsulate data groupings for locations with salamanders *vs.* random locations.

We observed that when comparing upland habitat environmental conditions between salamander and random locations, salamanders favoured cooler substrates (*V* = 45, *p* = < 0.001; Wilcoxon signed rank test with a continuity correction), with a mean substrate temperature of 18.6 ± 1.0 °C being favoured by salamanders compared to random locations of 20.3 ± 1.1 °C (Table 2; Fig. 3). No other upland habitat environmental variables were statistically or biologically significant between salamander and random locations, including upland soil pH (*V* = 229.0, *p* = 0.0747; Wilcoxon signed rank test with a continuity correction), soil moisture (*t*_25_ = −0.863, *p* = 0.397; paired *t*-test on log_10_ transformed data), and canopy cover (*V* = 318.5, *p* = 0.170; Wilcoxon signed rank test with a continuity correction) (Table 2).

We also examined upland environmental variables, except for plant percent coverage by type, with a PCA where PC1 and PC2 explained 68.52% of the total variance (Table S1). After visualising the data points and 95% confidence ellipses for PC1 and PC2, there appeared to be a large overlap (Fig. 3). A Wilcoxon rank sum test with a continuity correction was completed to compare PC1 and PC2 scores for locations with and without salamanders, which demonstrated no significant differences between location types (*W* = 441, *p* = 0.111 and *W* = 266, *p* = 0.133, respectively).

**Figure 4:**
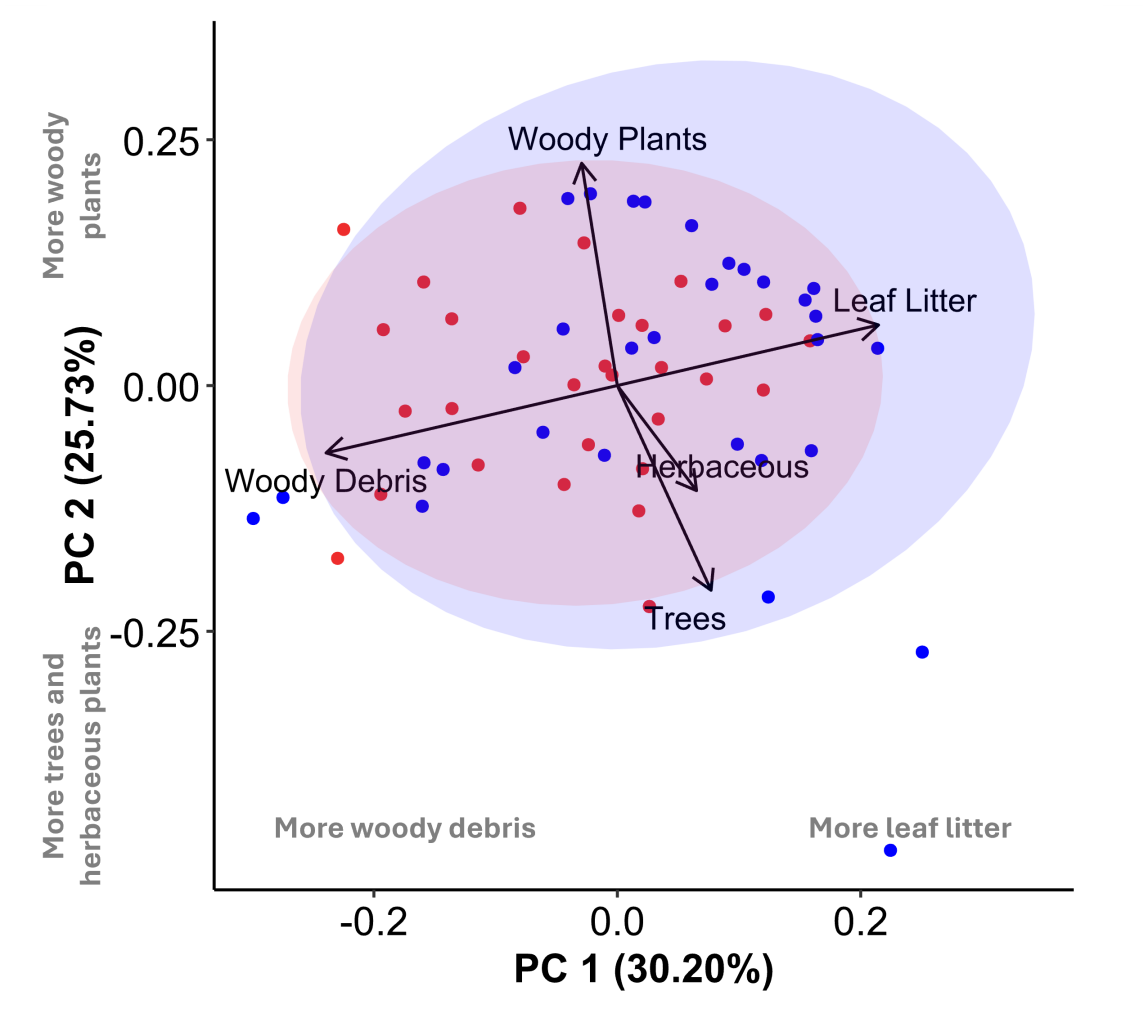
Upland plant type principal component plot for points where Four-toed Salamanders (*Hemidactylium scutatum*) were found (red) and paired random locations (blue). Principal component one (PC1) is plotted along the *x*-axis with its corresponding PC scores, explaining 30.20% of the total variance. PC1 is dictated primarily by wooden debris and leaf/needle litter with loadings of −0.708 and 0.635, respectively. Principal component two (PC2) is plotted along the *y*-axis with its corresponding PC scores, explaining 25.73% of the variance. PC2 is dictated primarily by woody plants, herbaceous plants (herbaceous), and trees with loadings of 0.668, −0.316, −0.616, respectively. The 95% confidence ellipses encapsulate data groupings by type (salamander and random locations).

For plant percent coverage by type, PC1 and PC2 explained 55.93% of the total variance (Table S2). After visualising the data points and 95% confidence ellipses for PC1 and PC2, there appeared to be a large overlap (Fig. 4). A two-sample *t*-test and a Wilcoxon rank sum test with a continuity correction were completed to compare PC1 and PC2 scores for locations with and without salamanders, respectively. These tests demonstrated no significant differences between location types (*t*_60_ = −0.051, *p* = 0.959 and *W* = 541, *p* = 0.398, respectively).

## Discussion

This study confirms that the second population of Four-toed Salamanders (*Hemidactylium scutatum*) discovered in New Brunswick has successful egg production and juvenile recruitment occurring at this site, despite its disturbed ecosystem. This finding is supported by observations of five clutches of eggs with and without female presence observed, eight juveniles, and an observed sex ratio that can support ongoing reproduction. The instance where two clutches of eggs were found in the same *Sphagnum* spp. hummock with one female may be a result of the Four-toed Salamander communal nesting behaviour (*i.e.,* where a gravid female will lay their eggs with those of a guarding female and leave; Harris & Gill, 1980; Harris et al., 1995; Harris, 2008). This behaviour is thought to occur when a female does not have enough energy to take care of the eggs (*i.e.,* not enough fat stores to sustain herself as the eggs develop; Harris, 2008). Alternatively, we may have overlooked the other female if she was in that hummock. It is also worth noting that females with low body conditions may skip reproductive years all together (Harris & Ludwig, 2004). Demographically, body condition did not differ between sexes. The standard error around the mean SMI values for females and males overlaps the mean of the opposite sex (females: 0.53 ± 0.03 g; males: 0.54 ± 0.03 g) which suggests a good deal of variability within the population overall. In terms of the microhabitats these salamanders selected within the environment, our findings did not support our primary hypothesis (*i.e.,* individuals were targeting specific microhabitats in an otherwise unfavourable landscape) but did support aspects of our alternate hypothesis (*i.e.,* individuals had access to suitable habitat within the broader landscape that suited their thermal and hydric needs) suggesting they are not selecting particular microhabitats at this site. In short, we found no statistically significant differences between microhabitat conditions where a salamander has chosen to be and at random paired locations, except for moss hummock slope in fen habitats and substrate temperature in upland habitats (Table 2). These findings align with Chalmers & Loftin (2006) who include bank slope in a model predicting nest presence, and Crawford & Semlitsch (2008) who reported soil temperature as a key predictor for salamander abundance (*i.e.,* lower temperatures correlate with higher salamander occupancy). Taken as a whole, these findings point to a population surviving in a landscape that is distinct from the less urbanised habitats that are commonly associated with this species (Wood, 1955; Kilpatrick, 1997; Chalmers & Loftin, 2006; Vitale, 2013; King & Richter, 2022) because the microhabitats in this landscape still meet their needs.

Our examination of the population status and demographics of the Riverview population suggests that the population is currently producing successful offspring, with an observed demographic structure that could allow for continued persistence. There is evidence that human activity and habitat alterations can significantly reduce reproductive success and juvenile recruitment in amphibian populations (Lowe, 2012; Keely et al., 2015; Bodinof-Jachowski & Hopkins, 2018). Currently, we do not have enough data to determine if reproductive success here is changing over time. Finding five clutches of eggs in our fens and juveniles within the upland forest suggests population recruitment is occurring and that the habitat supports their reproductive needs; however, this is a low clutch number compared to the approximately 11 nests per site observed in Kentucky, USA, by King & Richter (2022) and the 25–160 nests per site observed in Tennessee, USA, by Ferguson & Hamed (2024). The strong presence of females in the population, with an adult sex ratio of 1:1.5 (M:F) derived from 1:2.3 (M:F) in 2024 and 1:0.9 (M:F) in 2025, is also a positive sign for successful population recruitment moving forward. The 1:0.9 (M:F) ratio during the non-female biased survey year (2025) closely aligns with the 1:1 ratio we would expect (Fisher, 1930) and suggests there is not a particular threat for one sex or the other. To ensure a non-female bias in future Four-toed Salamander population studies, we suggest, like Blanchard & Blanchard (1931), searching in autumn under cover objects between September–November during breeding season (*i.e.,* when males and females are equally as active and when males are more easily identifiable *via* secondary sex characteristics; Bishop, 1941; Rucker et al., 2021). Sex ratios for this species are rarely reported within the literature; however, some early work suggested that a more southern Four-toed Salamander population appeared male-biased in juvenile specimens (*i.e.,* 1:0.76 (M:F); Blanchard, 1935). In another plethodontid species with a range that overlaps the Four-toed Salamander (*i.e.,* the Eastern Red-backed Salamander), sex ratios in forest habitats tend to not significantly differ from a Fisherian predicted 1:1 (M:F) ratio (Fisher, 1930; Jaeger et al., 1995; Gillette, 2003; Riedel, Russell & Ford, 2012). Further, no juveniles were observed in 2025; however, this year had an extremely warm and dry spring and summer which may have lowered larval and juvenile survival. Yet it is also possible that by chance alone we simply did not overturn the cover objects that had juveniles underneath them in 2025. Continued surveying at the site is required to determine if there remains to be indicators of low juvenile recruitment post-drought.

Sexual size dimorphism in body length (SVL) was observed with males tending to be longer, which has not previously been reported in Four-toed Salamanders. An opposite trend, in SVL sexual dimorphism, has been documented by Blanchard & Blanchard (1931; Michigan, USA) and for total length in Gilhen (1984; Nova Scotia, Canada) and Kilpatrick (1997; West Virginia, USA). It is possible that there is geographic variation in the length at which Four-toed Salamanders mature, and thus to fully understand trends in size differentiation, the length at which Four-toed Salamanders become sexually mature at our site would need to be verified (*e.g.*, *via* dissection). To our knowledge, there are no other studies on Four-toed Salamanders that examine the differences in length between the two sexes; thus, more research is needed to know if the species exhibits sexual dimorphism regarding SVL. Qualitatively, the demography and evidence of recruitment suggests that the Riverview population has a range of age, size, and sex classes, and should be viewed as an established population. Yet, longer term monitoring is needed to understand population ecology trends and health.

A factor in understanding a population’s conservation status (*i.e.,* their degree of imperilment), beyond their reproductive success, is the question of population size and density. Although we did not conduct a population size estimate, owing to a single recapture being identified, we report an estimated relative abundance of 67 salamanders between 2024 and 2025; however, given we presumably did not capture every individual within the site, there are likely many more. Nevertheless, using a conservative estimate of relative abundance, we calculated a minimum density of 2.12 individuals/ha. Our study collected count data on the number of individuals encountered during our surveys, which occurred above-ground. Our above-ground survey effort prevented us from locating all salamanders at the site and this is corroborated by our low number of recaptures (*n* = 1). This is simply a consequence of our non-destructive sampling approach that cannot sample salamanders that are present below the substrate or within moss, logs, or cover objects as to not negatively impact these important habitat features. Thus, it is likely we are only scratching the surface on how many salamanders there really are present at the site, and additional surveys over the long-term would be beneficial to refine our understanding of their population ecology at this site. The closest documentation of relative abundance with similar survey methods is based out of the Halifax Regional Municipality, Nova Scotia, Canada, where 337 individuals were found on roads in various locations (Moore, 2011), which is a higher individual count than all individual encounters in New Brunswick to date (McAlpine, 2013; Christiansen et al., 2026; and this study). Several studies completed in the United States have individual counts ranging from 42–487 individuals at various sites (Chalmers & Loftin, 2006; Vitale, 2013; King & Richter, 2022). Overall, these abundance estimates are predominantly higher than what we observed at the Riverview site (*i.e.,* 67 unique individuals and five clutches of eggs). One explanation could be that this site, despite having the capacity for juvenile recruitment, is unable to sustain a higher population density, potentially because of urbanisation (*e.g.*, habitat degradation). Alternatively, Riverview’s low detection numbers may be reflective of the species’ population density at the northern extent of their range. Given we do not know the population’s relative abundance or relative density at the Fundy National Park site, beyond the qualitative ‘low density’ description in Woodly & Rosen (1988), we cannot determine if the Riverview estimates are within the regional norm. Future work should examine population abundance and density fluctuations in both this and the Fundy National Park population for provincially relevant comparisons.

Although the extremely low recapture rate (*i.e.,* a single individual) prevented our ability to generate a population estimate, the lone recaptured individual provided insight into salamander movement. The recaptured adult male found first on 19 August 2024 and then on 1 October 2025 travelled at least 52.8 m within the 408 days between each occurrence location. To our knowledge, this is the first report of the distance a male Four-toed Salamander can travel within their upland habitat. Other records of Four-toed Salamander movement appear to document the distance females and males are from the edge of nesting locations ranging from 5.5–72 m (Pouliot & Bastien, 2009). Home range sizes for Four-toed Salamanders remain unknown. The closest comparison we can make regarding home range size in the autumn months (when Four-toed Salamanders are not migrating) is with Eastern Red-backed Salamanders (*Plethodon cinereus*) that can have home ranges of 1–24 m^2^ (Kleeberger & Werner, 1982; Mathis, 1991; Gade et al., 2023). However, because Eastern Red-backed Salamanders do not have an aquatic life stage that requires migration, the overall home range of the Four-toed Salamander is likely larger. Many knowledge gaps regarding the spatial ecology of Four-toed Salamanders exist that merit further investigation.

Across many of the environmental factors we measured, we found that the Riverview population is fitting within expected norms (Table 2). Overall, comparisons to literature values (Table 2) support the assertion that the Riverview site contains suitable nesting, breeding, and feeding habitat. A key finding from our comparisons between salamander and random locations was that, for the most part, they are not meaningfully different. The broad and consistent similarity between salamander occupied and salamander unoccupied random locations appear to indicate that salamanders are not being forced into small microhabitat pockets that align with more pristine conditions described in the literature (*e.g.*, Chalmers & Loftin, 2006; Wahl, Harris & Nelms, 2008; Casebere & Lodato, 2011). While cool, deciduous forest is a consistent requirement for most terrestrial plethodontid salamanders (*e.g.*, Eastern Red-backed Salamanders, Two-lined Salamanders; Christie, 2024) the Riverview site holds the additional feature (*i.e.,* a bog/fen habitat) that Four-toed Salamanders need to persist. Four-toed Salamanders may be less of a habitat specialist when it comes to environmental microhabitat conditions, in relation to other terrestrial plethodontid salamanders, but more of a specialist at the macrohabitat level (*i.e.,* requiring a fen/bog next to a forest rather than minimally disturbed ecosystems). While there are some examples of Four-toed Salamander populations near anthropogenic development (*e.g.*, Vitale, 2013; Wade et al., 2023) the Riverview population, surrounded by anthropogenic landscapes (*e.g.*, roads, parking lots, and suburban spawl), would appear to be an extreme of the urbanised populations published to-date. Four-toed Salamanders here seem to be able to persist within this human-modified habitat because their needs for temperature, humidity, canopy coverage, pH, vegetation, and cover objects have been met. In short, this urbanised greenspace appears to function as a refuge amidst accelerating habitat alterations and development. The presence of bogs/fens with shallow wetlands and dense nesting substrate may be more limited in urban environments; however, these bogs/fens should not be overlooked when surveying for additional Four-toed Salamander populations. For researchers and wildlife managers looking to locate future Four-toed Salamander populations, we suggest not overlooking disturbed areas, so long as the essential environmental components these salamanders need are present.

Although generalist taxa that can adapt to quickly changing landscapes have the best chances of survival in urbanised areas (Ishitani, Kotze & Niemelä, 2003; Sorace & Gustin, 2009; Ducatez et al., 2018), there is evidence that specialist taxa can survive in these areas when essential habitat components, critical for their survival, are retained. Our findings point to the idea that these salamanders could be a species that can withstand degrees of urbanisation, if certain habitat features, like a functional fen surrounded by adjacent upland forest habitat (Bai et al., 2019), are met. Similar scenarios have been observed in a few other salamander species, such as Blue-spotted Salamanders (*Ambystoma laterale*) and Eastern Tiger Salamanders (*Ambystoma tigrinum*) in Illinois (Vanek, King & Glowacki, 2019), Chignahuapan Splayfoot Salamanders (*Chiropterotriton orculus*) in Mexico (Serrano et al., 2022), and Eastern Red-backed Salamanders in Atlantic Canada (Christie, 2024). The ability of Four-toed Salamanders, and some other salamander species, to withstand and persist within a certain degree of urbanisation provides some hope that humanity’s need to convert and alter landscapes will not inevitably eliminate salamanders. However, when urban development causes populations to become fragmented by roads, housing, and other major developments, like what is seen with our population of salamanders, there is high risk of a loss of gene flow and connectivity that can inevitably plummet genetic diversity, fitness, and population persistence overall (Couvet, 2002; Reed & Frankham, 2003; Wade et al., 2025). Minimising connectivity loss is important as salamanders provide important services for their ecosystems (*e.g.*, top-down effects regulating invertebrate compositions; Wyman, 1998; Laking et al., 2021). Ensuring that habitats holding Four-toed Salamanders do not become fragmented will reduce the risk of inbreeding and population declines (Blomqvist et al., 2010; Benson et al., 2019; Williams et al., 2021). Ongoing urban development must minimise ecosystem harm by maintaining existing greenspaces, connectivity between greenspaces, and ensuring that species have what they need to thrive (Casebere & Lodato, 2011; Gallo et al., 2017; Zuñiga Palacios et al., 2021). In the case of Four-toed Salamanders, it is also necessary to minimise disruptions to the hydrology of the wetlands that are critical for nesting and the larval life stage, while also maintaining connection to adjacent forests with moderate to dense canopy cover and a forest floor rich in cover objects and moss to maintain their microhabitat needs (Casebere & Lodato, 2011). Moving forward, it is important to ensure that Four-toed Salamander nesting habitats are not separated from forests by new roads, where salamanders are present, as roads can cause population declines (Ochs, Swihart & Saunders, 2024). Luckly, separation by roadways between nesting and forest habitats is not the case for our population. As human populations continue to grow, more species will be forced to find new ways of living in anthropogenically modified habitats, but with proper planning and care, many of these species can still thrive.

## Conclusions

Our study provides foundational information regarding a newly discovered population of Four-toed Salamanders in New Brunswick, Canada—a species, now found in only two distinct locations in the province. We found evidence of breeding, juvenile recruitment, and normal demographic parameters. Our observations suggest that the Riverview population is using microhabitats that align with expected norms compared to other studied populations. Through our comparative approach we have determined that this population is persisting at this urbanised site, not because they are locked into rare or limited environmental requirements, but rather that much of the Riverview site appears to be habitable for them. Overall, data deficiency hinders conservation globally, as limited research guiding conservation action prevents many species from receiving adequate protections (De Ornellas, Milner-Gulland & Nicholson, 2011; Borgelt et al., 2022), especially for amphibians (Howard & Bickford, 2014). By studying sensitive, and understudied, species with cryptic lifestyles that can be enveloped and isolated by human development, such as Four-toed Salamanders, we can better understand what must be done to avoid potential population declines.

##  Supplemental Information

10.7717/peerj.21370/supp-1 Supplemental Information 1 Supplementary materials. Additional information about the principal component analyses in the main manuscript, study site, survey types, body condition estimation curves, locations of Four-toed Salamanders in 2024 and 2025, and visualizations of fen environmental variable data between locations with and without salamanders.

## Additional Information and Declarations

### Field Study Permissions

The following information was supplied relating to field study approvals (*i.e.*, approving body and any reference numbers): All individuals were handled under a scientific permit from the Government of New Brunswick (Permit #: SP24-006). To prevent the spread of disease between individuals and populations, the most recent decontamination guidelines were followed (Canadian Herpetofauna Health Working Group, 2017). Permissions for private property access were provided by the New Brunswick Department of Transportation, Riverview High School, and the Town of Riverview.

### Data Availability

The following information was supplied regarding data availability: The data and code are available at Open Science Framework: - https://doi.org/10.17605/OSF.IO/ED8NF
- Leal, A. M. J., Christie, G. A., Riley, J. L., & Baxter-Gilbert, J. (2026, April 28). Data and Code from “A specialist surrounded by suburbia: The ecology of a newly described Four-toed Salamander population in New Brunswick”. https://doi.org/10.17605/OSF.IO/ED8NF.
