# Supplementary material for: A specialist surrounded by suburbia: the ecology of a newly described Four-toed Salamander population in New Brunswick
Source: PeerJ. 2026 Jun 8;14:e21370. doi: 10.7717/peerj.21370 (PMC13256048; doi:10.7717/peerj.21370)
Supplement: Supplemental Information 1 — Additional information about the principal component analyses in the main manuscript, study site, survey types, body condition estimation curves, locations of Four-toed Salamanders in 2024 and 2025, and visualizations of fen environmental variable data between locations with and without salamanders. [file peerj-14-21370-s001.pdf]

A specialist surrounded by suburbia: the ecology of a newly described Four-toed Salamander population in New Brunswick

Ashton M. J. Leal, Georgia A. Christie, Julia L. Riley, James Baxter-Gilbert

Supplementary Materials

The supplementary materials contain additional information about the principal component analyses in the main manuscript, study site, survey types, body condition estimation curves, locations of Four-toed Salamanders in 2024 and 2025, and visualizations of fen environmental variable data between locations with and without salamanders.

Tables

Table S1: Principal component analysis loadings of principal components (PC) 1–4 for upland environmental variables. The eigenvalues and the percent of the variance explained is displayed for each principal component.

Variables	PC1	PC2	PC3	PC4
Substrate temperature	NA	0.892	NA	0.448
Soil pH	0.567	-0.198	-0.668	0.440
Soil moisture	-0.626	-0.333	NA	0.704
Canopy cover	0.535	-0.233	0.741	0.332
Eigenvalues	1.612	1.129	0.771	0.488
% of variance explained	40.30	28.23	19.27	12.21

Table S2: Principal component analysis loadings of principal components (PC) 1–5 for plant percent coverages recorded within a 2500 cm^2 quadrat at locations with salamanders and their paired, random points. The eigenvalues and the percent of the variance explained is displayed for each principal component.

**Variables**	**PC1**	**PC2**	**PC3**	**PC4**	**PC5**
Leaf/Needle Litter	0.635	0.182	0.444	0.203	0.571
Wooden Debris	-0.708	-0.202	0.245	0.114	0.621
Trees	0.228	-0.616	NA	-0.732	0.182
Woody Plants	NA	0.668	-0.385	-0.514	0.365
Herbaceous Plants	0.192	-0.316	-0.771	0.383	0.350
Eigenvalues	1.510	1.286	1.117	0.791	0.296
% of variance explained	30.20	25.73	22.34	15.82	5.916

Figures

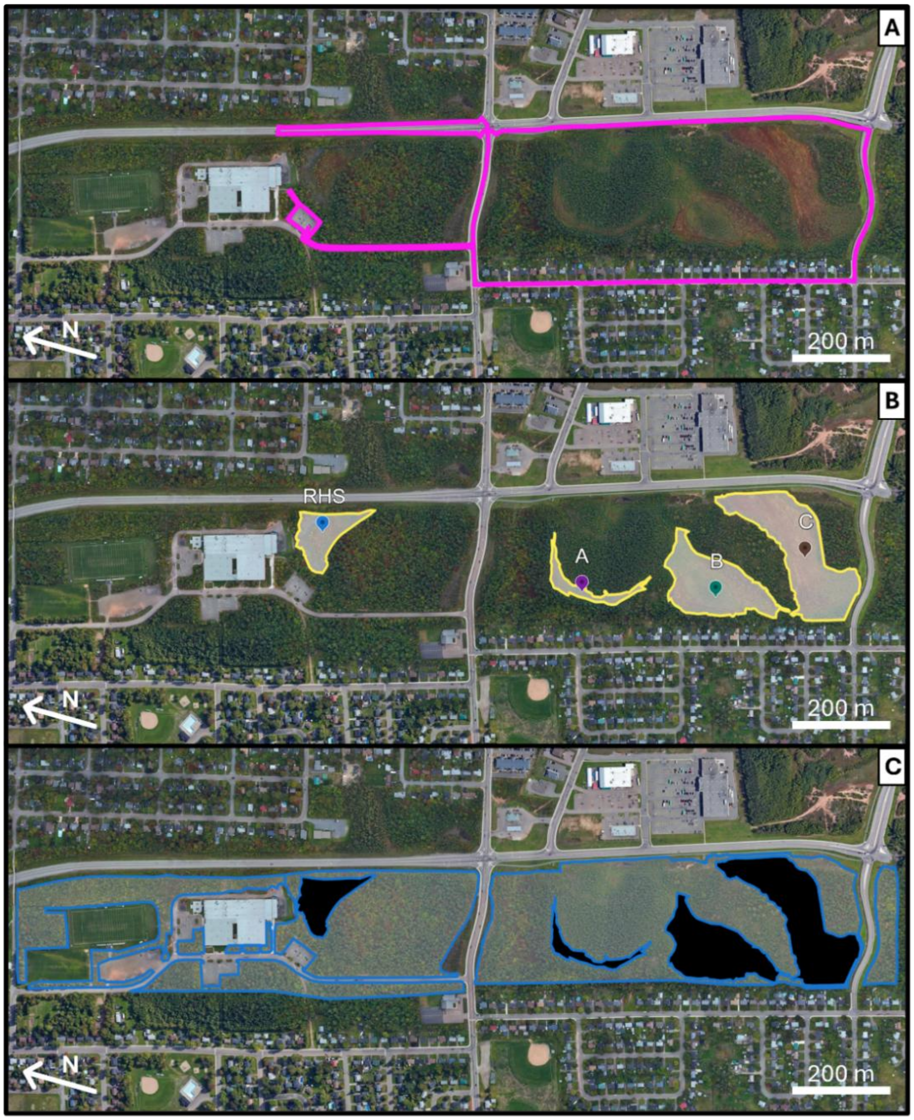

Figure S1: Survey routes and areas covered while searching for Four-toed Salamanders (*Hemidactylium scutatum*) in Riverview, New Brunswick, Canada. All mapping was created in Google Earth® (accessed on 1 April 2025; the map includes data from Google® and imagery from 22 September 2017) at the study site in Riverview, New Brunswick. The arrow points towards north for all maps. **(A)** Road survey route along the road (~3,951.84 m) starting and ending at Riverview High School. Surveys were conducted after dusk during precipitation events (e.g., ranging from mist to heavy rain) when the roads were wet, and when the air temperature was above 5 °C. **(B)** Fen nearest Riverview High School (RHS) and fens A, B, and C, that were surveyed 4 – 5 times per week between 15 May – 28 June 2024. The RHS fen is ~ 0.85 ha and was fully surveyed three times during nesting season. Fen A, B, and C are ~ 0.4, 2.01, 2.86 ha, respectively, and were fully surveyed twice. **(C)** Upland survey areas (~ 31.55 ha) that were surveyed 4 – 5 times per week between 19 June – 11 November 2024 and 28 August – 11 November 2025.

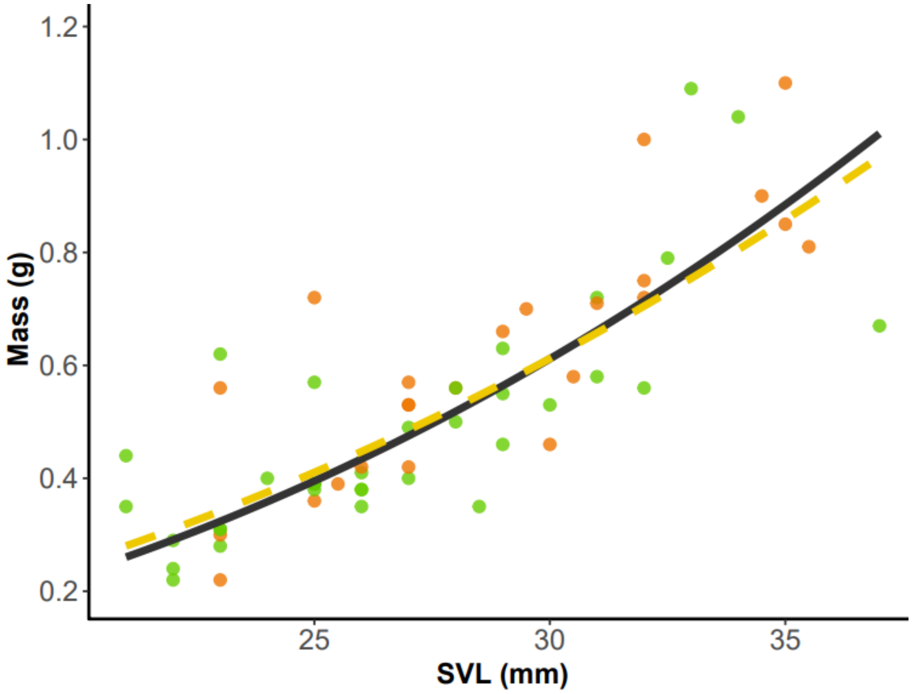

Figure S2: Mass (g) plotted against snout-vent length (SVL) in mm for Four-toed Salamander (*Hemidactylium scutatum*) females (green, *n*=35) and males (orange, *n*=24). Lines of best fit show the predicted scales mass index estimation curves using ordinary least squares (yellow/gold dashed line) (Peig and Green 2009) versus robust regression (black solid line) (Maronna et al. 2019). Points represent raw data of Four-toed Salamanders. We visually inspected this plot to assess fit and determined that the robust regression estimation method appeared to better follow the trend observed in the data.

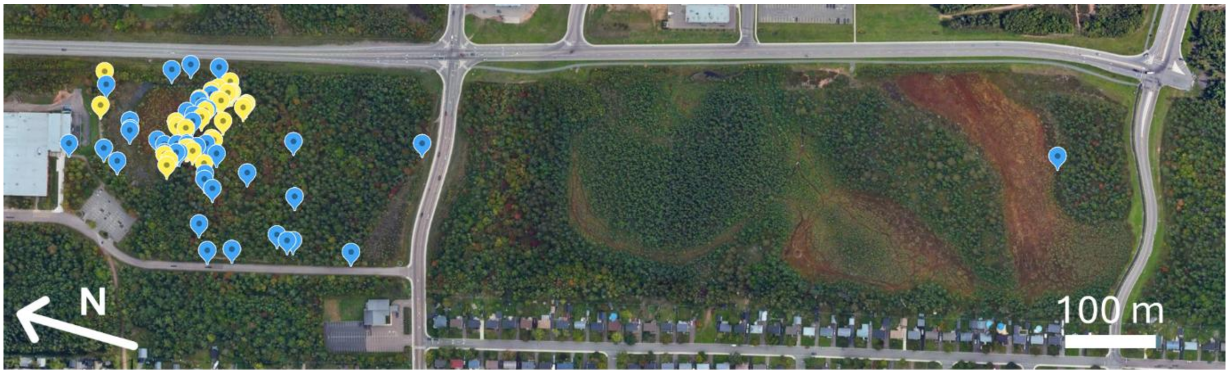

Figure S3: Occurrences of Four-toed Salamanders (*Hemidactylium scutatum*) and egg masses in Riverview, New Brunswick, Canada. Blue markers represent individuals and/or egg masses located in 2024 and yellow markers represent individuals located in 2025. This map was created in Google Earth® (accessed 19 November 2025; the map includes data from Google® and imagery from 22 September 2017).

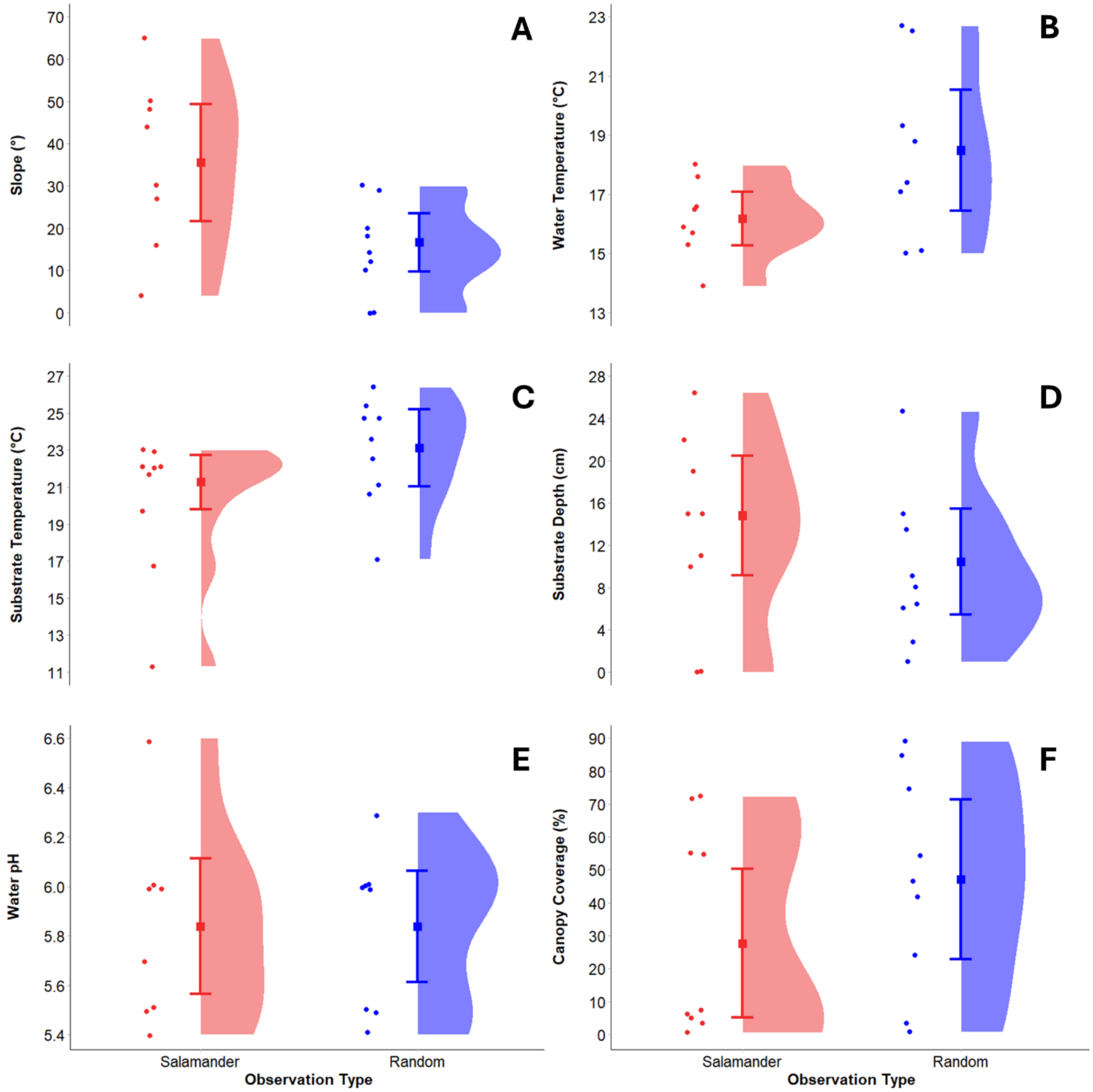

Figure S4: Fen environmental variable plots for points where Four-toed Salamanders (*Hemidactylium scutatum*), eggs, or both, were found (red) and paired random locations (blue). The square points are means, the error bars are 95% confidence intervals, the points on the left of the error bars are raw data, and half violin plots are on the right of the error bars. The y-axes represent **(A)** moss hummock slope in degrees, **(B)** water temperature (°C), **(C)** substrate temperature (°C), **(D)** depth of the substrate vegetation (cm), **(E)** water pH, and **(F)** canopy coverage (% coverage).

References

Maronna R, Martin D, Yohai V, Salibian-Barrera M. 2019. *Robust statistics: theory and methods (with R), 2nd Edition.* Hoboken: John Wiley & Sons.

Peig J, Green AJ. 2009. New perspectives for estimating body condition from mass/length data: the scaled mass index as an alternative method. *Oikos* **118**(12): 1883-1891. DOI: 10.1111/j.1600-0706.2009.17643.x
